# Household decision-making and the mental well-being of marriage-based immigrant women in South Korea

**DOI:** 10.1371/journal.pone.0263642

**Published:** 2022-02-22

**Authors:** Eunji Lee, Soo In Kim, Kyunghee Jung-Choi, Kyoung Ae Kong

**Affiliations:** 1 Department of Preventive Medicine, College of Medicine, Ewha Womans University, Seoul, Korea; 2 Kyungpook National University Law School, Daegu, Korea; 3 Department of Psychiatry, College of Medicine, Ewha Womans University, Seoul, Korea; 4 Department of Occupational and Environmental Medicine, College of Medicine, Ewha Womans University, Seoul, Korea; University of West London, UNITED KINGDOM

## Abstract

**Objective:**

We assessed the association between household decision-making and mental well-being among Asian immigrant women residing in Korea. We also investigated if the impact varies by the regional origin and examined potential factors for joint decision-making.

**Methods:**

We conducted a cross-sectional study using the Korean National Survey of Multicultural Families 2015 and logistic regression. We analyzed data from 11,188 married immigrant women ages 20 to 59 who were originally from East Asia or Southeast/South Asia and co-living with their spouses. We defined households as joint-decision, wife-decision, or husband-decision based on who decides how living expenses are spent. Mental well-being encompassed a depressive mood for two consecutive weeks, and life and marital satisfaction.

**Results:**

After controlling for covariates, we showed that immigrant women in husband-decision households were more likely to have depressive mood (odds ratio [OR] 1.26, 95% confidence interval [CI]: 1.14–1.38), poorer life satisfaction (OR 1.49, 95% CI: 1.24–1.78), and poorer marital satisfaction (OR 1.81, 95% CI: 1.47–2.22) than women in joint-decision households. Immigrant women in wife-decision households had a similar but slightly lower odds of poor mental well-being. This association was less prominent for Southeast/South Asian origin than East Asian origin, while the age-adjusted prevalence rates of poor mental well-being among them were higher than their East Asian counterparts. Factors that restricted the odds of husband-decision did not necessarily guarantee wife-decision.

**Conclusions:**

This study suggests one-sided decision-making can be a risk factor for immigrant women’s poor mental well-being, while joint decision-making is protective. Differences across regional origins suggest domestic decision-making might be a less important predictor for mental well-being in immigrants more occupied with adapting to the host society. Factors for joint decision-making should be addressed to improve the mental well-being of immigrant women.

## Introduction

It has been reported that gender inequality affects women’s mental and physical health across diverse populations [1]. One aspect of gender inequality was pointed out as household decision-making power [2–4]. Explained as a woman’s ability to control her own living environment, it is also conceptualized as “autonomy,” “empowerment,” “position within household,” etc. [5]. Women with low domestic decision-making power have limited access to health care [6], including dietary plan and maternal health resources [7]. They participate less in fertility behavior, such as family planning [8–10] and modern contraceptive use [11–13]. They also have worse health outcomes, even after adjustment for socioeconomic status [14].

However, especially when it comes to mental health, heterogeneous results were drawn out [15]. While low power for the wife was associated with higher stress or depression due to lack of control over living conditions [3, 16], higher power could imply the wife’s burden within the household and, therefore, was a predictor for poor mental health [17]. To better capture the meaning of women’s participation in decision-making, some studies have conceptualized joint decision-making between spouses as containing more equal gender beliefs and practices [18, 19].

International marriage migration is reported to contain unequal gender relations between husband and wife because of the gendered nature of a commercial marriage market [20]. However, there is a scarcity of research on immigration and domestic decision-making. Studies that investigated women in households where both spouses migrated to another country reported that immigration encourages the wife’s personal autonomy and therefore is associated with higher life satisfaction [21, 22]. When the couples were separated due to the husbands’ migration, their wives’ increased autonomy was associated with more depressive symptoms [23]. Yet, whether household decision-making among marriage-based immigrant women is associated with mental well-being is understudied.

Marriage-based immigrant women are subject to prejudice and discrimination [24]. In South Korea (hereafter, Korea), men’s international marriage rates increased in the 1990s [25]. Probably due to sociodemographic changes such as declining birth rate, population concentration in cities, and increase in women’s education and employment, population shrinkage and gender imbalance have continued to deepen in Korean rural areas, and a shortage of partners for unmarried men in rural areas triggered the migration of foreign brides from less developed countries [26, 27]. Most marriages were arranged by agencies that helped Korean men choose their potential brides and immigrant women resolve their economic difficulties from their home countries [28, 29]. Studies of marriage migrants in Korea have revealed that exposure to a new culture and lifestyle may affect poor mental health [29–32].

In this context, we anticipated that immigrant women’s low control of living conditions would be associated with poor mental well-being. This association was expected to vary between East Asian and Southeast/South Asian immigrants residing in Korea based on distinct immigration history and the economic and cultural backgrounds of their birth countries. For instance, while East Asians have geographical, ethnical, and demographic commonalities with Koreans, Southeast and South Asian immigrants are distinguished even in appearance and socio-economic circumstances [33–35]. As for the migratory process, most of the Southeast/South Asian women started to come to Korea 20 years ago, just for the sake of marriage and economic satisfaction as “foreign brides” [25], whereas East Asian immigrants’ history can be traced back to the early twentieth century, when Koreans migrated to China and Japan for refuge- or job-seeking under Japanese rule and war [36].

This study aimed to examine whether marriage-based immigrant women participating in any one-sided household decisions rather than joint decision are at a greater risk of depressive mood and of poor life and marital satisfaction. Additionally, we investigated if the impact varies by regional origin and identified potential factors associated with household decision type—joint-, wife-, or husband decision.

## Materials and methods

### Data source and study subjects

We used the cross-sectional dataset from the Korean National Survey of Multicultural Families (NSMF) 2015 released by the Ministry of Gender Equality and Family and by Statistics Korea. The NSMF includes a nationally representative sample of multicultural families residing in South Korea and has been conducted every three years since 2009.

The targets of NSMF 2015 are the family members of multicultural families (marriage migrants, their spouses, and offspring) and non-marriage migrants who naturalized to Korean citizenship. The sampling frame was based on the 2010 Korean Census, which identified 278,294 multicultural families in 3,487 administrative districts nationwide. Among 1,045 districts extracted by a systematic cluster sampling, 27,120 households were selected using stratified systematic sampling. The strata consist of two types of administrative district (urban and rural) and 14 nationality categories. For the data collection, in-person interviews were conducted by NSMF trained personnel. A total 17,849 households were contacted and responded to the 2015 survey (response rate of 65.8%), and 14,218 households included immigrant women ages 20 to 59.

Among the 14,218 women, we identified 12,552 participants who were both married and living with their spouse (1,804 not married, 1,389 missing information on whether they are living with their spouse; these frequencies were not counted exclusively). We additionally excluded those from regions other than East/Southeast/South Asia, and whose nationality at birth was not South Korean. Finally, the study population was confined to 11,188 immigrant women who were aged 20 to 59, married, co-living with their spouse, and from East Asia or Southeast/South Asia.

This study was approved by the Seoul Hospital of Ewha Womans University Medical Center Institutional Review Board (IRB number: SEUMC 2019-06-008). All data were fully anonymized before public release by the Ministry of Gender Equality and Family, hence the IRB waived the requirement for informed consent.

### Measurements

As a measure of household decision-making, we used a single item about the financial decision of household purchases because it was the only question applied to all subjects among three items of decision-making in the survey (child education, changes in their own employment/job status, and family purchases), regardless of having children, job-seeking activity, and current nationality/visa status. It has also been the most frequently used to reveal the marital relationship in many studies. Thus it was defined by the survey question, “Who decides to spend the living expenses in your household?” Survey participants answered using a 5-point Likert scale (on a scale of 1 to 5: *I decide entirely*; *I decide mostly*, *and spouse helps*; *we decide fairly*; *spouse decides mostly*, *and I help; and spouse decides entirely*), and responses of 1 or 2 were categorized as *wife*, 4 or 5 as *husband*, and 3 as *joint-decision making households*.

Mental well-being consisted of depressive mood, life satisfaction, and marital satisfaction, as evaluated by self-report. Depressive mood was measured by a survey question: “During the past year, have you ever felt so sad or hopeless that you were unable to do daily activities for two consecutive weeks or more?” The possible responses were *never experienced*, *have experienced sometimes*, *often*, or *very often*. For the regular categorization, which was consistent with the assessment of the sixth Korea National Health and Nutrition Examination Survey (KNHANES 2015), all answers except for *never experienced* were classified as “depressive.” For the conservative categorization, “depressive” included only *often* and *very often*. Life satisfaction and marital satisfaction were measured by the following survey questions, respectively: “Considering your overall daily life, how satisfied are you with your current life?” and “How satisfied are you with your relationship with your spouse?”. The answers were rated on a 5-point scale and categorized as “poor life/marital satisfaction” (*unsatisfied* and *very unsatisfied*) or “good life/marital satisfaction” (*very satisfied*, *satisfied*, and *fair*).

Sociodemographic variables were used as covariates in analyses of the association between mental well-being and household decision-making and between decision-making and potential factors. These variables included: age (grouped as 20–29, 30–39, 40–49, and 50–59), educational level (middle school education or less, high school education, and college education or more), years of stay in Korea (five years or fewer, six to nine years, and 10 years or longer), residential area based on governmental administration (urban or rural), and number of children (no children versus one child or more). Regional origin was dichotomized into East Asia (including Korean Chinese and China, Japan, Taiwan, and Hong Kong) and Southeast/South Asia (including Vietnam, the Philippines, Thailand, Cambodia, and other Southeast/South Asian countries) based on the strata design of the NSFM survey and the United Nations Standard Country or Area Codes for Statistical Use [34]. Monthly average household income was grouped as less than 1 million won, 1–2 million won, 2–3 million won, 3–4 million won, and 4 million won or more (1 million won approximates to 884 USD, considering the average currency rate in 2015 (1,131 won for 1 USD) issued by The Economic Statistic System, Bank of Korea). Monthly average personal income was categorized as no income, less than 1 million won, and 1 million won or more. Korean language proficiency was assessed by the summed score on four domains—speaking, listening, reading, and writing—for which the respondents could answer using a 5-point scale. The total score was classified as fluent (4–8), fair (9–15), or poor (16–20).

In the analysis of potential factors associated with decision-making, we included as additional covariates subjective socioeconomic status (dichotomized into low versus middle/high) and average daily conversation time with spouse (less than 1 hour and 1 hour or longer). Subjective socioeconomic status was asked about through the following question, “Which social status do you think your family hold?” and was included to incorporate immigrant women’s sense of relative deprivation or perception of her social status that are hardly indicated by variables such as income or education. To measure attitudes toward gender roles, participants were asked on the survey, “How much do you agree with the following statement? ‘A man’s role is to earn money, and a woman’s is to take care of the family.’” The 5-point Likert scale responses from *I agree very much* (1) to *I don’t agree at all* (5) were dichotomized into traditional (responses 1 to 3) and egalitarian (responses 4 and 5).

### Statistical analysis

The descriptive characteristics of the immigrant women across the three types of decision-making households and two regional origins were presented as frequencies and percentages. We estimated the age-adjusted prevalence of depressive mood and of poor life and marital satisfaction by types of decision-making household, using the 10-year age distribution interval for the total study population. Logistic regression analysis was used to determine the association, with adjustment for sociodemographic variables such as age, education, residential area, household income, personal income, years of stay in Korea, regional origin, Korean language proficiency, and number of children. Additionally, we conducted a stratified analysis by regional origin. In this analysis, the model included the interaction term of household decision-making and regional origin in addition to the control variables used in the first analysis.

We also assessed potential factors associated with household decision-making. We conducted a multinomial logistic analysis, where the odds were formed by comparing wife-decision or husband-decision to joint-decision. The reverse OR is interpreted as the odds of joint decision relative to wife or husband decision. All statistical analyses were conducted with SAS statistical software (version 9.4; SAS Institute; Cary, NC).

## Results

### Characteristics of the study subjects

The sociodemographic characteristics of the study subjects are presented in Table 1. Immigrant women in joint- and wife-decision households were more likely to come from East Asia (56.6% and 64.7%, respectively), while those in husband-decision households were more likely to come from Southeast/South Asia (66.4%). Overall, immigrant women in the joint- and wife-decision households were likely to be older, have more of their personal income, live in urban areas, be more fluent in Korean, and have lived longer in Korea. By regional origin, immigrant women from East Asia were older, more educated, more fluent in Korean, had higher household and personal income, and lived longer in Korea than their Southeast/South Asian counterparts.

**Table 1:** Characteristics of study subjects (N = 11,188).

			Household-decision			Regional Origin	
			Joint-decision	Wife-decision	Husband-decision	East Asia	Southeast/South Asia
			(n = 3838)	(n = 3261)	(n = 4089)	(n = 5659)	(n = 5529)
		N	(%)^a^	(%)	(%)	(%)	(%)
Regional Origin							
	East Asia	5659	56.6	64.7	33.6		
	Southeast/South Asia	5529	43.4	35.3	66.4		
Decision-making							
	Joint decision	3838				38.4	30.1
	Wife decision	3261				37.3	20.8
	Husband decision	4089				24.3	49.1
Age (year)							
	20–29	3335	24.5	19.8	42.8	8.9	51.3
	30–39	4109	38.1	38.3	34.2	37.8	35.7
	40–49	2633	26.5	29.4	16.1	35.6	11.2
	50–59	1111	10.9	12.5	7.0	17.8	1.9
Education							
	≤Middle school	3203	25.0	24.4	35.4	20.0	37.5
	High school	4879	43.4	46.4	41.6	46.9	40.2
	≥ College	3106	31.6	29.2	23.0	33.1	22.3
Household income (million won^b^)							
	<1.00	472	3.8	4.8	4.2	3.6	4.9
	1.00–1.99	2323	19.3	20.6	22.3	17.8	23.8
	2.00–2.99	3965	34.0	33.6	38.3	32.9	38.0
	3.00–3.99	2573	24.5	23.2	21.5	23.8	22.2
	≥4.00	1855	18.5	17.9	13.8	21.9	11.1
Wife’s personal income (million won)							
	No income	5554	43.7	44.0	59.7	46.7	52.7
	< 1.00	2152	19.1	20.2	18.7	18.4	20.1
	≥ 1.00	3482	37.2	35.8	21.6	34.9	27.2
Residential area							
	Rural area	6816	38.9	35.3	42.3	34.1	44.2
	Urban area	4372	61.1	64.7	57.7	65.9	55.8
Number of children							
	No children	2560	26.6	21.1	20.9	29.3	16.4
	1 child	4190	36.2	35.8	39.9	33.4	41.6
	≥2 children	4438	37.2	43.1	39.2	37.3	42.1
Korean proficiency							
	Poor	726	5.1	3.9	9.9	4.9	8.1
	Fair	5995	51.0	43.0	64.4	36.4	71.2
	Fluent	4467	43.8	53.2	25.7	58.8	20.7
Years of stay in Korea							
	≤5	3314	25.3	18.1	42.8	16.5	43.1
	6–9	3599	32.9	30.5	32.8	28.5	36.0
	≥10	4275	41.8	51.3	24.4	55.1	21.0

^a^Difference of the characteristics among three groups of household-decision and among two groups of regional origin were all <0.001.

^b^1 million won = approximately 884 USD, considering the average currency rate in 2015 (1,131 won for 1 USD).

### Mental well-being of immigrant women by household decision-making

Table 2 reveals that, compared with the immigrant women in joint-decision households, those in the wife- and husband-decision households had poorer mental well-being. The age-adjusted prevalence rates of depressive mood and poor life satisfaction were 39.3% and 9.0% in husband-decision households, 36.6% and 9.1% in wife-decision households, and 33.4% and 6.1% in joint-decision households.

**Table 2:** The association between household decision-making and poor mental well-being among immigrant women.

Poor mental well-being		Joint-decision-household	Wife-decision-household	Husband-decision-household
		(N = 3838)	(N = 3261)	(N = 4089)
Depressive mood––regular categorization	N (%)^a^	1264 (32.9%)	1157 (35.5%)	1658 (40.6%)
	Prevalence (SE)^b^	33.4 (0.8)	36.6 (0.9)	39.3 (0.8)
	OR (95% CI)^c^^d^	1 (ref^e^)	1.14 (1.03–1.26)	1.26 (1.14–1.38)
Depressive mood––conservative categorization	N (%)	219 (5.7%)	209 (6.4%)	319 (7.8%)
	Prevalence (SE)	5.9 (0.4)	6.8 (0.4)	7.4 (0.4)
	OR (95% CI)	1 (ref)	1.13 (0.93–1.38)	1.27 (1.05–1.52)
Poor life satisfaction	N (%)	247 (6.4%)	315 (9.7%)	337 (8.2%)
	Prevalence (SE)	6.1 (0.4)	9.1 (0.5)	9.0 (0.4)
	OR (95% CI)	1 (ref)	1.42 (1.19–1.70)	1.49 (1.24–1.78)
Poor marital satisfaction	N (%)	165 (4.3%)	225 (6.9%)	279 (6.8%)
	Prevalence (SE)	4.2 (0.4)	6.6 (0.4)	7.2 (0.4)
	OR (95% CI)	1 (ref)	1.55 (1.26–1.92)	1.81 (1.47–2.22)

^a^Differences in prevalence among 3 groups were all <0.001.

^b^Prevalence (%) was 10-year age-standardized with the total study subjects.

^c^OR: odds ratio, 95% CI: confidence interval.

^d^ORs were adjusted for age, education, residential area, household income, wife’s personal income, years of stay in Korea, regional origin, Korean language proficiency, and number of children.

^e^ref: reference level of each variable.

Even after controlling for sociodemographic factors, we found that women in husband-decision households were more likely to have depressive mood (OR 1.26, 95% CI: 1.14–1.38), poorer life satisfaction (OR 1.49, 95% CI: 1.24–1.78), and poorer marital satisfaction (OR 1.81, 95% CI: 1.47–2.22) than women in joint-decision households. Immigrant women in wife-decision households were also more likely to have depressive mood (OR 1.14, 95% CI: 1.03–1.26), poorer life satisfaction (OR 1.42, 95% CI: 1.19–1.70), and poorer marital satisfaction (OR 1.55, 95% CI: 1.26–1.92) than women in joint-decision households.

Prevalence and ORs differed by regional origin (Table 3). The age-adjusted prevalence rates of depressive mood, conservatively categorized depressive mood, and poor marital satisfaction were higher among women from Southeast/South Asia, while poor life satisfaction was higher in women from East Asia. The association of mental well-being with household decision-making was more prominent for women from East Asia than for women from Southeast/South Asia. Among East Asian immigrants, women in husband-decision households were more likely to have depressive mood (OR 1.42, 95% CI: 1.22–1.64), poorer life satisfaction (OR 1.89, 95% CI: 1.48–2.40), and poorer marital satisfaction (OR 2.29, 95% CI: 1.70–3.07) than women in joint-decision households. ORs of women in wife-decision households to joint-decision households were lower yet significant for depressive mood (OR 1.18, 95% CI: 1.03–1.35), poorer life satisfaction (OR 1.56, 95% CI: 1.25–1.96), and poorer marital satisfaction (OR 1.81, 95% CI: 1.37–2.38). For Southeast/South Asian immigrants, the association of domestic decision-making with poor mental well-being in both husband- and wife-decision households was mostly not significant, but women in husband-decision households reported higher odds for depressive mood (OR 1.15, 95% CI: 1.01–1.31) and poorer marital satisfaction (OR 1.42, 95% CI:1.07–1.88) than those in joint-decision households. The magnitude of the association of household decision-making and mental well-being in Southeast/South Asian women was significantly smaller than that of East Asian women (*p* for interaction<0.05 for regularly categorized depressive symptoms, poor marital satisfaction in S1 Table).

**Table 3:** The association between household decision-making and poor mental well-being among immigrant women—stratified by regional origin^a^.

Poor mental well-being		East Asia			Southeast/South Asia		
		Joint-decision-household	Wife-decision-household	Husband-decision-household	Joint-decision-household	Wife-decision-household	Husband-decision-household
		(N = 2173)	(N = 2111)	(N = 1375)	(N = 1665)	(N = 1150)	(N = 2714)
Depressive Mood––regular categorization	N (%)^b^	612 (28.2%)	677 (32.1%)	502 (36.5%)	652 (39.2%)	480 (41.7%)	1156 (42.6%)
	Prevalence (SE)^c^	28.4 (0.9)	31.6 (1.0)	34.3 (1.0)	38.4 (1.0)	41.7 (1.1)	44.3 (0.9)
	OR (95% CI)^d^^e^	1 (ref^f^)	1.18 (1.03–1.35)	1.42 (1.22–1.64)	1 (ref)	1.09 (0.93–1.27)	1.15 (1.01–1.31)
Depressive mood––conservative categorization	N (%)	96 (4.4%)	120 (5.7%)	92 (6.7%)	123 (7.4%)	89 (7.7%)	227 (8.4%)
	Prevalence (SE)	4.8 (0.5)	5.7 (0.5)	6.4 (0.5)	6.9 (0.5)	7.8 (0.6)	8.5 (0.5)
	OR (95% CI)	1 (ref)	1.27 (0.97–1.68)	1.48 (1.10–2.01)	1 (ref)	1.01 (0.76–1.35)	1.13 (0.90–1.43)
Poor life satisfaction	N (%)	145 (6.7%)	221 (10.5%)	166 (12.1%)	102 (6.1%)	94 (8.2%)	171 (6.3%)
	Prevalence (SE)	6.5 (5.5)	9.4 (8.3)	9.3 (8.2)	5.8 (0.5)	8.7 (0.6)	8.6 (0.5)
	OR (95% CI)	1 (ref)	1.56 (1.25–1.96)	1.89 (1.48–2.40)	1 (ref)	1.23 (0.91–1.66)	1.12 (0.87–1.46)
Poor marital satisfaction	N (%)	87 (4.0%)	153 (7.3%)	118 (8.6%)	78 (4.7%)	72 (6.3%)	161 (5.9%)
	Prevalence (SE)	3.9 (0.5)	6.4 (0.5)	6.9 (0.5)	4.4 (0.5)	6.9 (0.5)	7.4 (0.4)
	OR (95% CI)	1 (ref)	1.81 (1.37–2.38)	2.29 (1.71–3.07)	1 (ref)	1.26 (0.90–1.76)	1.42 (1.07–1.88)

^a^The full model is presented in S1 Table.

^b^Differences in prevalence among 3 groups were all <0.001.

^c^Prevalence (%) was 10-year age-standardized with the total study subjects.

^d^OR: odds ratio, 95% CI: confidence interval

^e^OR from the logistic regression model including age, education, residential area, household income, wife’s personal income, years of stay in Korea, Korean language proficiency, number of children, regional origin, and the interaction term of household decision-making and regional origin.

^f^ref: reference level of each variable.

### Factors associated with household decision-making

Table 4 shows a multinomial logistic analysis modeling the respective odds of wife- or husband-decision to joint-decision households. Women who stayed 10 years or more in Korea had higher odds of wife-decision households (OR 1.43, 95% CI: 1.23–1.67) than women who stayed five years or fewer, as well as lower odds of husband-decision households (OR 0.53, 95% CI: 0.46–0.62). Immigrant women from East Asia also had higher odds of wife-decision households (OR 1.34, 95% CI: 1.20–1.51) and lower odds of husband-decision households (OR 0.53, 95% CI: 0.47–0.59) than their Southeast/South Asian counterparts.

**Table 4:** Factors associated with the type of household decision-making.

		Wife decision relative to joint decision	Husband decision relative to joint decision
		OR (95% CI)^a^^b^	OR (95% CI)
Education^c^	≤Middle school	1 (ref^d^)	1 (ref)
	High school	1.00 (0.88–1.12)	0.80 (0.71–0.89)
	≥College	0.85 (0.74–0.97)	0.63 (0.55–0.71)
Household Income	<2.00	1 (ref)	1 (ref)
(million won^e^)	2.00–2.99	0.90 (0.79–1.03)	1.06 (0.94–1.20)
	≥3.00	0.88 (0.77–1.00)	1.09 (0.95–1.24)
Wife’s personal income	No income	1 (ref)	1 (ref)
(million won)	<1.00	0.98 (0.86–1.11)	0.76 (0.67–0.86)
	≥1.00	0.90 (0.80–1.02)	0.47 (0.42–0.53)
Subjective SES^f^	Low	1 (ref)	1 (ref)
	Middle and high	0.91 (0.82–1.02)	0.99 (0.89–1.11)
Residential Area	Rural area	1 (ref)	1 (ref)
	Urban area	1.17 (1.06–1.30)	0.97 (0.88–1.07)
Number of Children	No children	1 (ref)	1 (ref)
	≥1 child	1.34 (1.18–1.51)	1.13 (1.00–1.27)
Conversation with spouse	<1 hour	1 (ref)	1 (ref)
	≥1 hour	0.72 (0.65–0.79)	0.67 (0.61–0.74)
Korean proficiency	Poor	1 (ref)	1 (ref)
	Fair/Fluent	1.14 (0.90–1.44)	0.69 (0.57–0.83)
Years of stay	≤5	1 (ref)	1 (ref)
	6–9	1.15 (1.00–1.32)	0.69 (0.61–0.78)
	≥10	1.43 (1.23–1.67)	0.53 (0.46–0.62)
Gender role attitude	Traditional	1 (ref)	1 (ref)
	Egalitarian	1.12 (1.01–1.23)	0.93 (0.84–1.02)
Regional Origin	Southeast/South Asia	1 (ref)	1 (ref)
	East Asia	1.34 (1.20–1.51)	0.53 (0.47–0.59)

^a^OR, odds ratio; 95% CI, confidence interval.

^b^All ORs in the table were estimated from a multinomial logistic regression model, which used joint decision as the reference response category and compared other response categories (wife decision and husband decision) against it.

^c^The model included age and all variables listed in the first column of the table as independent variables.

^d^ref, reference level of each variable.

^e^1 million won = approximately 884 USD, considering the average currency rate in 2015 (1,131 won for 1 USD).

^f^SES, Socioeconomic status.

The women who had their personal income of 1 million won or more (OR 0.47, 95% CI: 0.42–0.53), compared with no income, or who were fair or fluent in Korean (OR 0.69, 95% CI: 0.57–0.83), compared with poor, had lower odds of husband-decision households but did not have significantly higher odds of wife-decision households.

More education was associated with lower odds of husband-decision and wife-decision households. Relative to a middle school education or less, a college education or more was associated with lower odds of husband-decision (OR 0.63, 95% CI: 0.55–0.71) and lower odds of wife-decision (OR 0.85, 95% CI: 0.74–0.97) households. That is, women with a college education had 1.60 times higher odds of a joint-decision than a husband-decision household and 1.18 times higher odds of a joint-decision than a wife-decision household. Likewise, women conversing more than an hour with spouse had higher odds of fair decision compared to husband-decision and to wife-decision (OR 1.48 and 1.40, respectively). These odds are reciprocal of those presented in Table 4 because of the reverse of reference of odds.

## Discussion

This paper indicated that household decision-making affects mental well-being among immigrant women in Korea and that differences by regional origin exist. There is a significant association between mainly one-sided household decisions and immigrant women’s depressive mood, poorer life satisfaction, and poorer marital satisfaction. This relationship is more prominent for migrants from East Asia than for their Southeast/South Asian counterparts. Furthermore, we identified factors associated with domestic decision-making, discovering that factors reported to the wife having some power operated in different ways when the wife’s decision and husband’s decision were addressed separately.

The prevalence of poor mental well-being was high among immigrant women residing in Korea and differed between East Asian and Southeast/South Asian immigrants. We assessed depressive mood twice by categorizing the answers regularly and conservatively. For the regular categorization, prevalence of depressive mood was 33.4% to 39.3%—about two to 2.5 times higher than that reported by Korean women in the sixth KNHANES. This finding is supported by a previous study that demonstrated migrants in Korea are at a high risk for depression because of acculturative stress [29]. The age-adjusted prevalence of depressive mood was higher in migrants from Southeast/South Asia than in those from East Asia (41.9% vs. 31.2%, respectively). It is possible that the mental well-being of Southeast/South Asian women is influenced by differences in cultural and economic background compared with their East Asian counterparts. A lack of Southeast/South Asian co-ethnics from the same country [26, 30, 37] who could possibly buffer the pressure of adapting to the host society might also result in poorer mental well-being.

We demonstrated that household decision type is associated with mental well-being among Asian marriage-based immigrant women in Korea. Not only husband-decision but also wife-decision households could be risk factors for poor mental well-being, while jointly made decision between spouses seemed to be the most protective against it. In previous studies, one-sided decisions of the wife were meant to indicate exclusive autonomy or power, yielding heterogeneous results. For instance, among rural Ugandan women with HIV, depression symptom severity was reduced in those with higher sexual relationship power relative to women with low power [3]. In contrast, high decision-making power was significantly associated with psychological distress among Ethiopian women initiating antiretroviral treatment [17]. Our study where both wife’s and husband’s decisions were associated with poor mental well-being supports previous reports that a woman who decides mostly by herself may feel burdened rather than empowered [5, 15]. As joint household decision contains more equal gender beliefs and practices [18, 19], it could be the most protective against poor mental well-being among marriage-based immigrant women in Korea. In the relationship between women’s mental well-being and household decision-making, joint decision was proven to be the most desirable, whereas wife’s decision didn’t seem to be protective against poor mental well-being due to its conflicting meanings; power or burden.

The impact of decision-making on mental well-being was more prominent in immigrant women from East Asia than in those from Southeast/South Asia. This difference might be attributable to the dissimilar routes of migration and the contrasting historical and cultural context of origin between the two groups, which can be traced back to the early twentieth century [38–40]. East Asian countries, especially China, have been receptive to Koreans then and allowed them to form a Korean diaspora ever since [36, 41]. The descendants, equipped with Chinese identity and nationality, started to migrate back to South Korea in the 1990s for various reasons—labor, marriage, education, etc. Immigrant women from Southeast/South Asia, on the other hand, were motivated mostly by marriage to enhance economic well-being rather than cultural and historical familiarity [33, 35]. The migration of these women was led by commercialized international marriage agencies in the early 2000s, when Korea’s demographic transition such as declining birth rate and shortage of young women in rural areas sparked a national need for foreign brides (25). The differences between the two regional groups are also proven in previous studies. As found in our study that Southeast/South Asian immigrant women were younger, less educated, less fluent in Korean, had lower household and personal income, and lived shorter in Korea, some Chinese and Japanese immigrant women identified themselves as being from relatively wealthy natal families, while most of the Vietnamese and Cambodian women were from poor families [42]. Their basic economic needs seem to have been met by transnational marriage migration to Korea. Therefore, achievement of this goal could have made household decision-making less influential for their mental well-being even though their higher prevalence of poor mental well-being might result from higher possibility of adjusting to the Korean society less comfortably, and experiencing more acculturative stress than their East Asian counterparts [43–45].

Furthermore, South Asian women’s perspectives on marriage could be tied to the cultural tradition of their home country. Family structure in Pakistan and North India takes the form of a multigenerational patrilineal household where women have to be obedient to their husbands and parents-in-law [18, 46]. The South Asian immigrant women’s cultural and historical background might cause them not to develop a sense of taking part in household decisions. Similarly, immigrant women from Vietnam, the Philippines, and other low-income countries tend to live outside large cities, where the majority of East Asian immigrants reside, meaning that Southeast Asian immigrants may live with either or both parents-in-law in these rural areas [30, 37, 42]. Since women in extended families are less likely to have autonomy than those in nuclear family households [47], Southeast Asian women might participate less in domestic decisions and even regard that situation as comfortable. They could prefer their husband’s decision-making over that of other family members or their own burdensome decisions. In contrast, East Asian immigrant women tend to prioritize a fairer marital relationship due to the rapid social and cultural transformation in women’s rights [48].

We compared the potential factors for joint decision-making. Most factors were working in different ways when one-sided decisions were addressed separately. Living longer in Korea and coming from East Asia versus Southeast/South Asia indeed increased women’s power—these factors also were associated with increasing wife-decision and lowering husband-decision situations. On the other hand, a wife having her own income and more fluency in Korean only lowered the likelihood of a husband-decision household and did not guarantee a wife-decision household. A somewhat surprising result was that education, reported to have a direct association with women’s autonomy in Nepal [49, 50] and spousal conversation lowered women’s household decision-making. Our findings suggest that these factors were associated with joint decisions relative to husband decision, but did not necessarily increase the likelihood of wife-decisions. Perhaps women with more education or their own income aspire for a fair conjugal relationship. Especially in marriage migration, which might encompass unequal gender relations by nature, women could expect a more supportive relationship rather than one-sided relationship power. Based on the decision-making power model, these results can also indicate that a Korean husband married to a foreign wife from a less developed country has such a strong power that the wife’s education or income merely balances the marital relationship rather than giving the wife more power.

There are potential limitations as each primary outcome (depressive mood, life satisfaction, and marital satisfaction) is anchored in just a single assessment item. As for the survey questions, which might be insufficient to encompass multiple aspects of depression and satisfaction, we thought they are at least straightforward and helpful to understand the mental well-being of immigrant women in Korea. The question used to measure depressive mood in the study has been used in the nationally representative survey (the Korea National Health and Nutrition Examination Survey) for the same purpose.

In our study, the measurement of household decision-making differed from that in other studies. In previous research, household decision-making was measured based on multiple items, such as major and minor household purchases, visits to relatives, health care of the wife or children, etc. [5, 6, 30]. Our study may have a limited view because only one decision-making item—the spending of living expenses was used. However, among multiple domains, decision-making about household purchases has been the most frequently used to reveal the marital relationship in previous studies [51–55]. Other different characteristics of our study are that we only used the wife’s sole report [18, 51], and that we defined joint decision based on a response *we decide fairly*, which was placed in the middle of the questionnaire asking by whom and to what extend household decision is made.

Some potential confounding variables used in previous studies, such as women’s age at marriage, age difference between spouses, husband’s education, and social support from family or the presence of ethnic friends or relatives, were not considered by our study. Women’s age at marriage was closely connected to years of stay in Korea because most of the immigrant women migrated at the time of marriage to their Korean husband. When age differences between spouses and husband’s education were additionally included in the analyses among subjects with these information, the results were not that different.

Another potential limitation is our study is based on a cross-sectional design, not a longitudinal one, therefore it would be improper to directly interpret the factors associated with mental well-being or household decision-making as determinant factors.

Despite these limitations, our study has important implications. We added evidence on the association between decision-making and mental well-being of marriage-based immigrant women, especially as measured by general questions on depressive mood and life/marital satisfaction, showing different impacts in the immigrant population from distinct backgrounds. This study suggested joint household decision-making’s helpful effect against poor mental well-being. In certain cases, factors affecting decision-making had opposite effects on the wife’s decision and on joint-decision-making. These results have implications for understanding and supporting mental well-being of marriage-based immigrant women residing in Korea. With regard to modifiable factors associated with the household decision type, supporting them to improve Korean proficiency or to have their own job and income if desired, and emphasizing or educating couples on spousal conversations and mutual respect of each other’s opinion on family finance (in multicultural family support centers throughout the country, etc.) could enhance joint-decision making of the marriage-based immigrant women’s households and be beneficial to their mental health, although this is not easy to assert due to the cross-sectional design and nature of this study.

## Conclusion

This study demonstrates that domestic decision-making affects mental well-being among Asian immigrant women in Korea, a marginalized population that lacks resources. Our findings suggest that joint decision-making might be protective while one-sided decision-making, even by the wife, could be a risk factor for depressive mood and for poorer life and marital satisfaction. Differences across regional origins of East and Southeast/South Asia were denoted, suggesting that domestic decision-making might be a less important predictor for mental well-being in immigrants more occupied with adapting to the host society. We offered an understanding of the potential factors for joint decision-making by revealing that they operated differently in husband- and wife-decision.

## Supporting information

S1 Data This is the dataset of all the data used for this manuscript. (SAV)

S1 Table The multiple logistic regression analysis for the association between household decision-making and poor mental well-being among immigrant women. (DOCX)
