# Supplementary material for: Household decision-making and the mental well-being of marriage-based immigrant women in South Korea
Source: PLoS One. 2022 Feb 22;17(2):e0263642. doi: 10.1371/journal.pone.0263642 (PMC8863252; doi:10.1371/journal.pone.0263642)
Supplement: S1 Table — (DOCX) [file pone.0263642.s002.docx]

S1 Table. The multiple logistic regression analysis for the association between household decision-making and poor mental well-being among immigrant women^a^

|  |  | Depressive  mood  –regular  categorization | Depressive  mood  –conservative  categorization | Poor life satisfaction | Poor marital satisfaction |
| --- | --- | --- | --- | --- | --- |
| Variables |  | OR (95% CI)^b^ | OR (95% CI) | OR (95% CI) | OR (95% CI) |
| **Household decision-making** | |  |  |  |  |
| East Asian | Joint-decision | 1 (ref^c^) | 1 (ref) | 1 (ref) | 1 (ref) |
|  | Wife-decision | 1.18 (1.03–1.35) | 1.27 (0.97–1.68) | 1.56 (1.25–1.96) | 1.81 (1.37–2.38) |
|  | Husband-decision | 1.42 (1.22–1.64) | 1.49 (1.11–2.01) | 1.89 (1.48–2.40) | 2.29 (1.71–3.07) |
| Southeast/ South Asian | Joint-decision | 1 (ref) | 1 (ref) | 1 (ref) | 1 (ref) |
|  | Wife-decision | 1.09 (0.93–1.27) | 1.01 (0.76–1.35) | 1.23 (0.91–1.66) | 1.26 (0.90–1.76) |
|  | Husband-decision | 1.15 (1.01–1.31) | 1.13 (0.90–1.43) | 1.12 (0.87–1.46) | 1.42 (1.07–1.88) |
| **Household Income**  **(million won**^d^**)** | ≥4.00 | 1 (ref) | 1 (ref) | 1 (ref) | 1 (ref) |
|  | 3.00–3.99 | 1.27 (1.12–1.45) | 1.02 (0.78–1.34) | 1.14 (0.86–1.49) | 1.38 (1.02–1.87) |
|  | 2.00–2.99 | 1.48 (1.30–1.67) | 1.14 (0.88–1.47) | 1.72 (1.34–2.22) | 1.79 (1.34–2.39) |
|  | 1.00–1.99 | 1.82 (1.58–2.09) | 1.68 (1.28–2.19) | 3.40 (2.63–4.40) | 3.20 (2.38–4.31) |
|  | <1.00 | 2.68 (2.15–3.34) | 3.20 (2.25–4.55) | 6.15 (4.40–8.61) | 3.91 (2.58–5.92) |
| **Education** | ≥College | 1 (ref) | 1 (ref) | 1 (ref) | 1 (ref) |
|  | High school | 1.05 (0.95–1.16) | 1.01 (0.84–1.21) | 0.98 (0.82–1.17) | 1.22 (0.99–1.50) |
|  | ≤Middle school | 0.99 (0.89–1.11) | 0.90 (0.73–1.12) | 1.15 (0.95–1.40) | 1.32 (1.05–1.66) |
| **Wife's personal income**  **(million won)** | No income | 1 (ref) | 1 (ref) | 1 (ref) | 1 (ref) |
|  | ≥1.00 | 1.17 (1.06–1.29) | 1.12 (0.92–1.36) | 1.76 (1.47–2.11) | 2.22 (1.82–2.71) |
|  | <1.00 | 1.26 (1.13–1.40) | 1.11 (0.91–1.35) | 1.54 (1.29–1.85) | 1.34 (1.07–1.67) |
| **Residential Area** | Rural area | 1 (ref) | 1 (ref) | 1 (ref) | 1 (ref) |
|  | Urban area | 1.29 (1.19–1.40) | 1.07 (0.92–1.25) | 1.14 (0.99–1.32) | 1.00 (0.85–1.18) |
| **Korean proficiency** | Fluent | 1 (ref) | 1 (ref) | 1 (ref) | 1 (ref) |
|  | Fair | 1.40 (1.28–1.54) | 1.23 (1.02–1.47) | 1.50 (1.27–1.76) | 1.57 (1.30–1.90) |
|  | Poor | 1.22 (1.02–1.45) | 1.36 (0.98–1.87) | 1.94 (1.44–2.61) | 2.21 (1.59–3.07) |
| **Number of children** | No children | 1 (ref) | 1 (ref) | 1 (ref) | 1 (ref) |
|  | ≥1 child | 1.30 (1.17–1.44) | 1.27 (1.03–1.57) | 1.62 (1.33–1.97) | 1.60 (1.28–2.00) |
| **Years of stay** |  | 1.00 (0.99–1.01) | 1.01 (0.99–1.02) | 1.02 (1.01–1.04) | 1.03 (1.01–1.05) |
| **Age (year)** |  | 1.00 (0.99–1.01) | 0.99 (0.98–1.00) | 1.02 (1.01–1.03) | 1.01 (0.99–1.02) |
| **Regional Origin** | East Asia | 1 (ref) | 1 (ref) | 1 (ref) | 1 (ref) |
|  | Southeast/South Asia | 1.37 (1.18–1.59) | 1.41 (1.05–1.91) | 0.82 (0.62–1.10) | 1.01 (0.72–1.41) |
| **Interaction of household decision-making and regional origin** | (wife-decision-household) | *p*=0.4269 | *p*=0.256 | *p*=0.2055 | *p*=0.1026 |
|  | (husband-decision-household) | *p*=0.0337 | *p*=0.1523 | *p*=0.004 | *p*=0.0193 |
|  |  |  |  |  |  |

^a^This is the full presentation of all the variables in the multiple logistic regression model in Table 3 other than those already presented.

^b^OR: odds ratio, 95% CI: confidence interval.

^c^ref: reference level of each variable.

^d^1 million won = approximately 884 USD, considering the average currency rate in 2015 (1,131 won for 1 USD).
